# Nationwide Stepwise Emergence and Evolution of Multidrug-Resistant *Campylobacter jejuni* Sequence Type 5136*,* United Kingdom

**DOI:** 10.3201/eid2507.181572

**Published:** 2019-07

**Authors:** Bruno S. Lopes, Norval J.C. Strachan, Meenakshi Ramjee, Anne Thomson, Marion MacRae, Sophie Shaw, Ken J. Forbes

**Affiliations:** University of Aberdeen, Foresterhill, Aberdeen, Scotland, UK

**Keywords:** *Campylobacter jejuni*, ST5136, multidrug resistance, clones, poultry, host reservoirs, endemic, broiler industry, antibiotics, antimicrobial drugs, antimicrobial resistance, Scotland, United Kingdom, bacteria

## Abstract

We examined whole-genome–sequenced *Campylobacter jejuni* and* C. coli *from 2012–2015 isolated from birds and human stool samples in North East Scotland for the presence of antimicrobial resistance genes. We found that sequence type (ST) 5136 (clonal complex 464) was the most prevalent multidrug-resistant strain of *C. jejuni* exclusively associated with poultry host reservoirs and recovered from human cases of campylobacteriosis. Tetracycline resistance in ST5136 isolates was due to a *tet*(O/32/O) mosaic gene, ampicillin resistance was conferred by G → T transversion in the −10 promoter region of *bla*_OXA-193_, fluoroquinolone resistance was due to C257T change in *gyrA,* and aminoglycoside resistance was conferred by *aac*. Whole-genome analysis showed that the strain ST5136 evolved from ST464. The nationwide emergence of ST5136 was probably due to stepwise acquisition of antimicrobial resistance genes selected by high use of β-lactam, tetracycline, fluoroquinolone, and aminoglycoside classes of drugs in the poultry industry.

*Campylobacter jejuni* and *C. coli* are the most common causes of bacterial foodborne gastroenteritis in the industrialized world (*1*). In the United Kingdom alone, *Campylobacter* is implicated in >500,000 cases, 80,000 medical consultations, and 200 deaths and costs the economy an estimated £1 billion annually (*2**,**3*). According to World Health Organization estimates, *Campylobacter*-related sequelae affect ≈1% of the worldwide population (*4*). In the United Kingdom, human infection has been associated with retail chicken meat products (55%–75% attribution); cattle and sheep have a secondary role and wild birds, pigs, and dogs minor roles (*5**–**7*). Most human *Campylobacter* infections are mild and self-limiting and resolve within a few days, but severe or prolonged infections can occur, particularly in the young, elderly, and immunocompromised patients with AIDS or other vulnerable categories for which therapeutic intervention may be warranted (*8*).

For clinical therapy of campylobacteriosis, antimicrobial drugs, such as erythromycin, are usually prescribed, but ciprofloxacin is advised in moderately severe cases of nonconfirmed gastroenteritis and for travelers’ diarrhea (*9*). In recent years, treatment with fluoroquinolones has been challenging because of an increasing prevalence of fluoroquinolone resistance in human *Campylobacter* isolates that led to the ban of the fluoroquinolone enrofloxacin for use in poultry in the United States in 2004 (*10*).

The Veterinary Medicines Directorate reports that in 2016 a total of 337 tons (17% decrease from 2015) of authorized veterinary antimicrobial drugs were sold in the United Kingdom. The sales of trimethoprim, sulphonamides, β-lactams, and aminoglycosides remained stable between 2012 and 2016, but notable reductions were observed for tetracycline (30%), macrolides (24%), and fluoroquinolones (29%) from 2015 to 2016 (*11*). Only enrofloxacin and difloxacin are licensed for poultry use; 0.5 metric tons of active ingredient were sold for use in poultry production in 2015 to the British Poultry Council, a national trade group that accounts for 90% of all broilers produced in the United Kingdom (*12*).

The main drivers for the acquisition of antimicrobial resistance (AMR) are selection pressure and the opportunity for horizontal gene transfer (*13*). Multidrug resistance has been defined as resistance to >3 classes of antimicrobial drugs and can occur by stepwise mutation or single plasmid acquisition of AMR genes (*14**,**15*). A report commissioned by the UK Food Standards Agency shows that drug-resistant *Campylobacter* species are becoming more prevalent; evidence shows increasing levels of resistance in bacteria from poultry meat (*16*), which makes up around half of all meat by weight purchased in the United Kingdom (33 kg/person) (*17*).

The aim of this study was to establish the evolutionary course of events that led to the emergence of a multidrug-resistant (MDR) organism by considering the interplay between selection pressures and genetic changes. Specifically, we investigated whether antimicrobial resistance to β-lactams, tetracyclines, fluoroquinolones, and aminoglycosides in the ancestral lineages of *C. jejuni* ST5136 (Figure 1) led to the emergence of a strain that is the causative agent of human campylobacteriosis in 1 of 20 cases in the United Kingdom (*18*).

**Figure 1:**
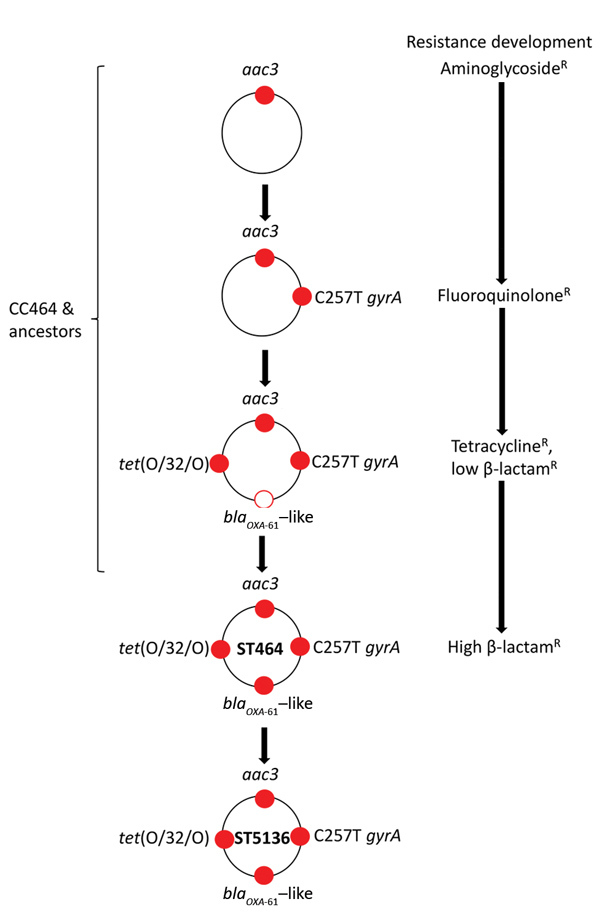
Stepwise sequential evolution of multidrug resistance in *Campylobacter jejuni* ST5136, Scotland. Red dots indicates resistance to antimicrobial drug as a result of genetic change or acquisition of resistance gene; white dot with red outline indicates acquisition of oxacillinase. Resistances are indicated as follows: *aac3,* aminoglycoside; C257T *gyrA,* fluoroquinolone; *bla*_OXA-61_–like, β-lactam (variant in ST5136 is OXA-193); *tet*(O/32/O), tetracycline. CC, clonal complex; OXA, oxacillin; R, resistant; ST, sequence type.

## Materials and Methods

### Bacterial Isolates and Genotyping

*Campylobacter* isolates for this study came from patients who had campylobacteriosis during 2012–2015 and from chicken and turkey retail meat samples. We incubated the meat samples at ambient temperature in enrichment broth for 1 h with occasional agitation and plated 100 μL of the broth on mCCDA plates (E & O Laboratories, http://www.eolabs.com). We then incubated the plates under microaerobic conditions at 37ºC for 48 h and extracted genomic DNA using the Wizard Genomic DNA Purification Kit (Promega, https://www.promega.com). Whole-genome sequencing was performed using HiSeq 2000 sequencer (Illumina, https://www.illumina.com) with 100 bped-end sequencing. We assembled FASTQ paired-end reads using Velvet (*19*) with a typical 50× coverage and assembled genome size of ≈1.6 Mbp. We uploaded the genomes to the Bacterial Isolate Genome Sequence Database (BIGSdb) and typed them using the 7-locus multilocus sequence typing (MLST) and whole-genome MLST (wgMLST) schemes (*20**,**21*).

### Antimicrobial Resistance Gene Determinants

We assessed AMRs in silico for clonal complexes (CC) 464 (parent CC of ST5136), CC353, CC354, and CC574 (n = 494, North East Scotland; n = 68, England). We determined the tetracycline resistance (*CAMP1698*) and *bla*_OXA-61_–like (*CAMP0265*) variants in accordance with BIGSdb (*20*). We assessed the flanking sequence of *bla*_OXA-61_–like for the G → T transversion in the –10 promoter region by exporting 100 bp flanking allele sequences in XMFA/concatenated FASTA formats from *C. jejuni*/*coli* PubMLST isolate database (*22*). We also screened isolates for *gyrA* and 23S rRNA V domain mutations, *bla*_OXA-184_–like, and presence of *ermA*, *emrB*, *ermC*, and *ermF* using BIGSdb and Resfams (*23**,**24*). We identified putative aminoglycoside resistance genes encoding N-acetyltransferases (AAC), O-adenyltransferases (ANT), O-phosphotransferases (APH), and streptothricin acetyltransferase (SAT) using Resfams (*25*).

### Genome Annotation and Phylogenetic Analyses

We chose 100 isolates with 1 *C. coli* (CC828) outgroup that were representative of the closely related clonal complexes CC464, CC353, CC354, and CC574 from BIGSdb. The group consisted of isolates from North East Scotland (n = 31), England (n = 68), and Estonia (n = 1). We annotated their genomes using RAST (*26*) and analyzed them by whole-genome multilocus sequence typing (wgMLST) using 1,643 Gundogdu loci (*27*). We constructed a phylogenetic tree in Bionumerics v. 7.6 (Applied Maths, http://www.applied-maths.com) using the topscore unweighted pair group method with arithmetic mean algorithm based on similarity of wgMLST loci. We constructed maximum-likelihood trees for aminoglycoside resistance determinants in MEGA7 (*28*).

### Antimicrobial Susceptibility Testing and Strain Stability

We determined antimicrobial susceptibility for 21 representative CC464 isolates with genotypic polymorphisms across the range of detected resistance markers. We used Mueller-Hinton agar with 5% defibrinated horse blood and 20 mg/L β-nicotinamide adenine dinucleotide for antimicrobial susceptibility testing using the disk diffusion method for ciprofloxacin (5 μg/disk), gentamicin (10 μg/disk), tetracycline (5 μg/disk), kanamycin (5 μg/disk), amikacin (30 μg/disk), tobramycin (10 μg/diskc), and streptomycin (30 μg/disk) (*29**,**30*). We determined MIC of ampicillin (AMP) by Etest (Oxoid, http://www.oxoid.com) and interpreted the MIC_AMP_ and disk susceptibility results using guidelines from the European Committee on Antimicrobial Susceptibility Testing and Clinical and Laboratory Standards Institute and interpreted them as described previously (*29*). We used the antimicrobial drug–susceptible isolate ARI3025 as a control. We subcultured the ST5136 isolate ARI4158 on antibiotic-free blood agar plate (ARI4158_20) 22 times to assess the stability of its antimicrobial resistance genes.

### Statistical Analyses

We calculated 95% CIs by 10,000 bootstrap iterations to assess if there were significant differences in clonal complexes or individual strains with respect to resistance to different antimicrobial drug categories or AMR resistance alleles. If the CIs did not overlap (e.g., between 2 groups for a particular factor), then we inferred the comparison to be statistically significant (p<0.05).

## Results

### Genomic Analyses of Resistance in CC464, CC353, CC354, and CC574

**Figure 2:**
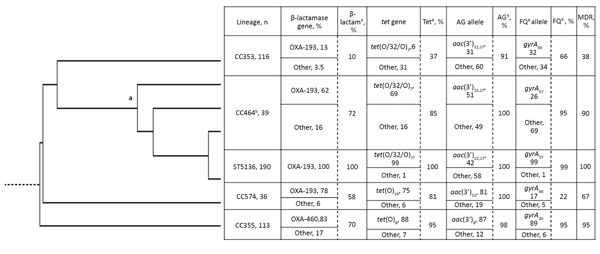
Whole-genome phylogenetic tree of *Campylobacter jejuni* CC464, CC353, CC574, and CC354 isolates, Scotland; a indicates CC464 root and b indicates CC464 isolates excluding ST5136. β-lactamase gene (*bla*_OXA-61_–like) indicates presence of abundant allele and other OXA genes; β-lactam^R^, resistant isolates (defined by −10 promoter mutation or presence of OXA-184–like gene). *tet* gene indicates presence of abundant tetracycline resistance allele and other alleles; Tet^R^, tetracycline-resistant isolates. AG allele indicates presence of abundant aminoglycoside allele and other alleles in a group of strains associated at the CC level; other indicates any other *aac3* resistance allele or a combination of the abundant allele along with a second *aac3*; AG^R^ indicates  aminoglycoside-resistant isolates. FQ allele indicates most abundant *gyrA* allele and other alleles that confer fluoroquinolone resistance; FQ^R^ indicates fluoroquinolone-resistant isolates. MDR is defined as resistance to >3 antimicrobial drugs. CC, clonal complex; MDR, multidrug resistant; OXA, oxacillin; R, resistant; ST, sequence type.

We compiled the metadata of 494 isolates from North East Scotland, including strain types and resistance genotypes (Appendix Table 1). This list includes the antimicrobial resistance genotype of isolates in CC464 (n = 229), CC353 (n = 116), CC354 (n = 113), and CC574 (n = 36) from across the United Kingdom. Of these, 97% of CC464 isolates were MDR, followed by 95% of CC354 and 67% of CC574; CC353 (38%) showed lower levels of antimicrobial resistance. Multidrug resistance in CC464 was significantly higher (p<0.05) than that for the other clonal complexes. All ST5136 isolates had genes or mutations conferring resistance to tetracycline, ciprofloxacin (all isolates except 1), and ampicillin. We observed >1 aminoglycoside resistance gene (*aac3* variants in ST5136 isolates with 75% (n = 8) showing phenotypic resistance to kanamycin (Table; Figure 2).

**Table:** Phenotypic evaluation of antimicrobial drug–resistant genotypes of *Campylobacter jejuni* ST5136 CC464 isolates, Scotland*

pubMLST ID	Isolate	Year	Source	*gyrA* C257T	CIP	OXA†	AMP	*tet* variant‡	TET	*aac3* variant	KAN	TOB
38459	ARI1530	2012	Human stool	T	R	A	S	*tet*(O)_80_	R	G1	R	S
39121	ARI2250	2013	Human stool	T	R	A	S	A	S	G1, G4	R	S
41320	ARI3377	2014	Human stool	T	R	OXA-465	R	*tet*(O/32/O)_7_	R	G1	R	S
40661	ARI3044	2013	Human stool	T	R	A	S	*tet*(O/M/O)_218_	R	G1	R	S
38785	ARI1899	2012	Human stool	T	R	A	S	*tet*(O)_13_	R	G1	R	R
38883	ARI2002_1	2012	Human stool	T	R	OXA-193 (G)	S	*tet*(O/32/O)_7_	R	G1	R	S
39844	C0525	2013	Turkey	T	R	OXA-193 (T)	R	*tet*(O/32/O)_7_	R	G1	R	S
42202	C1204	2014	Chicken	T	R	OXA-193 (T)	R	*tet*(O/32/O)_7_	R	G1, G4	R	R
40371	ARI2986	2013	Human stool	T	R	OXA-193 (T)	R	*tet*(O/32/O)_7_	R	G1, G4	I	S
48290	ARI4158	2015	Human stool	T	R	OXA-193 (T)	R	*tet*(O/32/O)_7_	R	G1, G4	I	S
42443	C1522	2015	Chicken	T	R	OXA-193 (T)	R	*tet*(O/32/O)_7_	R	G1, G4	R	S
58497	C0112	2010	Chicken	T	R	OXA-193 (T)	R	*tet*(O/32/O)_7_	R	G1	R	S
58473	ARI0533	2010	Human stool	T	R	OXA-193 (T)	R	*tet*(O/32/O)_7_	R	G1	R	S
41611	C0972	2014	Chicken	T	R	OXA-193 (T)	R	*tet*(O/32/O)_7_	R	G2	I	S
39810	C0469	2012	Turkey	T	R	OXA-193 (T)	R	*tet*(O/32/O)_7_	R	G1, G4	R	S
58159	ARI3975	2015	Human stool	C	S	OXA-193 (T)	R	*tet*(O/32/O)_7_	R	G1, G4	R	S
39411	ARI2623	2013	Human stool	C	S	OXA-193 (T)	R	A	S	G1	R	R
38522	ARI1599	2012	Human stool	T	R	OXA-193 (T)	R	*tet*(O/32/O)_7_	R	G1, G4	R	R
38475	ARI1546	2012	Human stool	T	R	A	S	*tet*(O/32/O)_7_	R	G1	R	R
48369	ARI3988	2015	Human stool	T	R	A	S	*tet*(O/32/O)_7_	R	G1	R	S
42114	ARI3830	2015	Human stool	C	S	OXA-193 (T)	R	*tet*(O/32/O)_7_	R	G1, G4	R	R
48290	ARI4158_20	2015	Human stool	T	R	OXA-193 (T)	R	*tet*(O/32/O)_7_	R	G1, G4	I	S

*Result based on disk susceptibility testing, except for ampicillin, for which MIC was estimated. R indicates MIC >128mg/L. All isolates were sensitive to amikacin, gentamicin, and streptomycin. *aac3,* 3-N-aminoglycoside acetyltransferase gene groups (G1–G4); AMP, ampicillin; CC, clonal complex; CIP, ciprofloxacin; I, intermediate; ID, identification; KAN, kanamycin; R, resistant; S, sensitive; ST, sequence type as determined by multilocus sequence typing; TET, tetracycline; TOB, tobramycin. †OXA-465 = bla_OXA-184_-like gene; A indicates absence of oxacillinase gene; (G) or (T) indicates guanine or thymine mutation at −10 promoter of *bla*_OXA-193._ ‡A indicates absence of tetracycline resistance gene.

We detected macrolide resistance in ARI2246 (ST2036, CC353), ARI2515 (ST3155, CC354), and ARI3316 (ST2438, CC354), all of which had A2075G mutation in the 23S rRNA V domain, observed by erythromycin disk diffusion assay. We found no mutations at 2074 and observed no resistance due to *erm* (erythromycin ribosome methylation) genes in any of the isolates.

Fluoroquinolone resistance determined by the *gyrA* (*CAMP0950*) C257T mutation was significantly higher in CC464 (226/229, 99%), the parent clonal complex of ST5136; CC353 (77/116, 66%); and CC354 (107/113, 95%) isolates, compared with CC574 (8/36, 22%) (p<0.05) (Figure 2; Appendix Table 1). β-lactam resistance was conferred primarily by the *bla*_OXA-61_–like (*CAMP0265*). *CAMP0265* allele 1 and 14 encoded the OXA-193 enzyme occurring in CC464, CC353, and CC574 isolates, whereas *CAMP0265* allele 38 (OXA-460) was significantly associated with CC354 group of isolates (p<0.05). ARI3377 (ST464) and ARI2874 (ST581) had *bla*_OXA-184_–like and lacked any *bla*_OXA-61_–like genes. CC353 isolates had relatively lower prevalence (19/113, 17%) of oxacillinase. Resistance to β-lactams as a result of a –10 promoter mutation upstream of the *bla*_OXA-61_–like or the presence of *bla*_OXA-184_–like was significantly greater in CC464 (217/229, 95%) and CC354 (79/113, 70%) compared with CC353 (11/116, 9%; p<0.05), whereas 21/36 (58%) of CC574 isolates were found to be resistant to β-lactams (p>0.05) (Appendix Table 1). Amino acid sequence identity revealed that OXA-193 (257 aa) and OXA-460 (253 aa) are 97.7% similar, which may be the result of environmental conditions or ecologic adaptations as listed previously (*31**,**32*).

**Figure 3:**
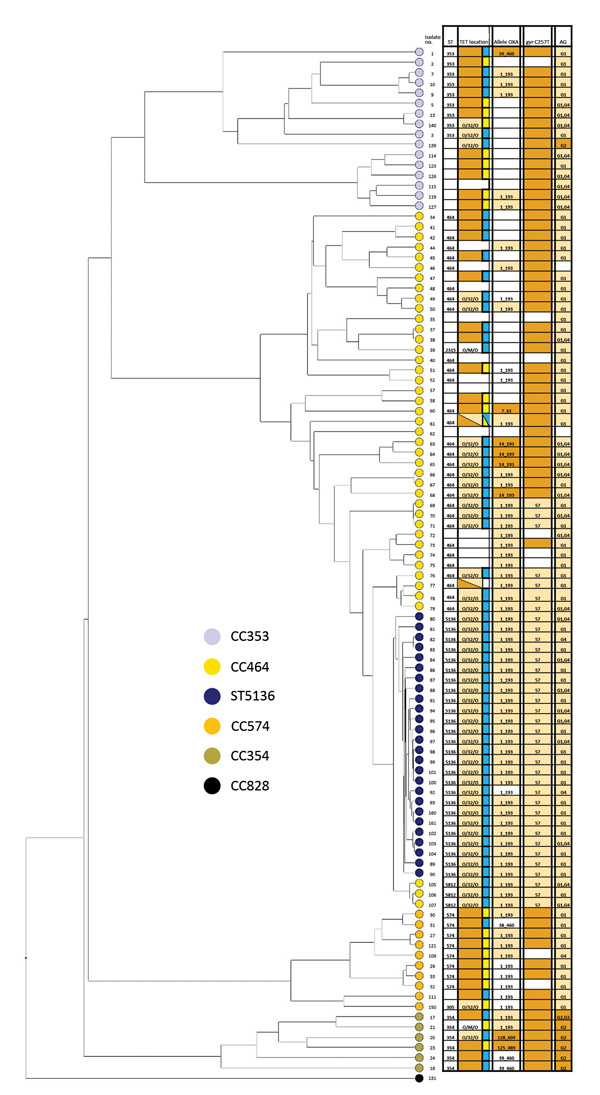
Whole-genome multilocus sequence typing of 100 selected isolates of *Campylobacter* *jejuni* from CC464, CC353, CC354, and CC574, Scotland. The tree was constructed using the UPGMA algorithm based on locus similarity. Isolate ID number indicated at branch end is linked to metadata in Appendix Table 2. Light orange boxes in the grid indicate variants associated with ST5136 isolates spread across the phylogeny; dark orange boxes denote resistance in individual isolates. Blue boxes represent tetracycline resistance in chromosome; yellow boxes, in plasmid. AG, aminoglycoside; CC, clonal complex; ID, identification; OXA, oxacillin; ST, sequence type; TET, tetracycline.

Tetracycline resistance was significantly higher in CC464 (221/229, 97%), CC354 (108/113, 96%), and CC574 (29/36, 81%) than in CC353 (43/116, 37%; p<0.05) (Appendix Table 1). The mosaic *tet*(O/32/O)_7_ was significantly associated with CC464, whereas the *tet*(O)_8_ and *tet*(O)_10_ were associated with CC354 and CC574 (p<0.05) (Figure 2). Of CC464 isolates, 93% (214/229) had the chromosomal *tet*(O/32/O)_7_–like disrupting *dcuC* (*CAMP1639*). Two ST464 clinical isolates, ARI3458 and OXC6581, were positive for both *tet*(O/32/O)_7_ and *tet*(O). In ARI3458, *tet*(O/32/O)_7_ was chromosomal and *tet*(O) was plasmidborne, but the location could not be determined in OXC6581 (Figure 3; Appendix Table 2). We observed that *tet*(O/32/O) alleles 7 and 22 and *tet*(O)_10_ occurred very rarely in CC353 isolates. We found the mosaic *tet*(O/32/O)_7_–like determinants on either plasmid or chromosome in CC353, CC354, CC574, and other clonal complexes but at a much lower frequency (Appendix Table 2). The *CAMP1698*_10 variant occurred in OXC8770, ARI1655, and ARI3389 (ST3015, CC574) and had 98.7% similarity (631/639) to *Streptococcus* phage_phi-m46.1 *tet*(O) sequence FM864213.1.

**Figure 4:**
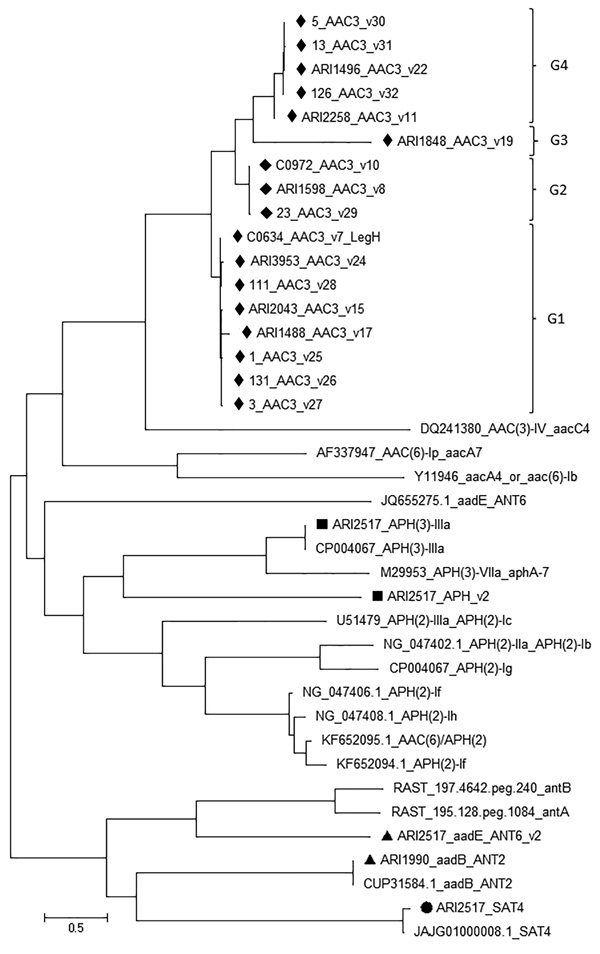
Maximum-likelihood tree of all aminoglycoside resistance determinants found in *Campylobacter* *jejuni* CC464, CC353, CC354, and CC574 isolates, Scotland. The tree was built using MEGA7 (https://www.megasoftware.net). The variants (v) listed in G1, G2, G3, G4 groups commonly occur in the 4 CCs. Scale bar indicates nucleotide substitutions per site. Aminoglycoside resistance gene variants identified in representative isolates of 4 CCs are shown by different symbols: black diamonds, *aac3*; black squares, *aph*; black triangles, *ant*; black circle, *sat.* CC, clonal complex.

The Resfams database identified aminoglycoside resistance determinants with lengths ranging from 96–263 aa. We did not assign allele numbers to determinants with polypeptide lengths of 96–169 aa and deemed them pseudogenes, whereas those with length >261 aa we identified as novel putative aminoglycoside resistance genes after BLAST query (Figure 4; Appendix Tables 1, 2). We identified a total of 17 *aac3* (groups G1–G4), 1 *aph* (O-phosphotransferase), and 1 *ant* aminoglycoside resistant gene. We grouped the *aac3* allelic variants as v7, v15, v17, v24–28, (G1); v8, v10, v29 (G2); v19 (G3); and v11, v22, v30–32 (G4) (Figure 4). We identified >1 *aac3* variant in all isolates and no *aac6′* enzymes. We identified streptothricin acetyltransferase (SAT_4), *aph* (v2), *aph3′-IIIa* (v1), and O-adenyltransferase (*aadE*/*ant6*, v2) in a single isolate of *C. jejuni* (ARI2517, ST2140/CC574). We identified the *aadE*/*ant6*, v2 in ARI2168 (ST400/CC353) also, and *aadB* in ARI1990 (ST2116/CC353) (Appendix Table 1). All CC464 isolates were positive for >1 *aac3* gene (Figure 2; Appendix Table 1, 2). The *aac3* allele v17 was significantly more common in CC464 compared with v8 and v22 that occurred in other clonal complexes (Figure 2).

### Antimicrobial Susceptibility Testing and Strain Stability

We performed susceptibility testing and corroborated 21 isolates of CC464 with genotypes predictive of resistant or sensitive phenotypes for different antimicrobial drugs (Table). Past studies show that disk diffusion correlated well to MIC determination and reliably distinguished between sensitive, intermediate, and resistant isolates of *Campylobacter* (*33*); hence, we used this method to assess and interpret genotypic data linked to isolate phenotype. Previous research has observed that the C257T *gyrA* genotype conferred ciprofloxacin resistance, G57T change in the −10 promoter of *bla*_OXA-61_-like led to an increase in ampicillin resistance, and all tetracycline resistance genes conferred resistance to tetracycline (Table) (*22*,*34*,*35*). We observed resistance to kanamycin in 18/21 *aac3*-positive isolates harboring alleles v22 and v17, indicating that these variants may have the ability to hydrolyze kanamycin (Table); this finding warrants further investigation. Most of the isolates displayed a tobramycin-sensitive phenotype, whereas all were sensitive to amikacin, streptomycin, and gentamicin (Table). We subcultured isolate ARI4158 (ST5136) 22 times on antibiotic-free blood agar to assess the stability of its antimicrobial resistance genes, but we found it to be resistant like the parent isolate.

### Phylogenetic Relationships

We constructed a phylogeny of 100 representative isolates of CC464 and associated clonal complexes using 1,643 wgMLST loci (Figure 3; Appendix Table 2). As predicted, ST5136 clustered within CC464; CC353, CC354, and CC574 isolates clustered, in that order, distal to CC464. ST5136 isolates were a single clonal MDR group closely related to the MDR isolates of ST5812 (CC464) reported in Guernsey in 2012 and now also found in England and Scotland (Figure 3). We observed the clustering of CC353, CC354, and CC574 around CC464 at the wgMLST level at which CC353 and CC354 strains had a high prevalence of ciprofloxacin resistance, whereas CC574 had a lower prevalence of ciprofloxacin resistance, our findings concur with those reported earlier (*36*). Strain ST6209 (isolate no. 72, resistant only to β-lactam) clustered around ST464 isolates (nos. 73, 74, 75) that had genotypes encoding low levels of resistance (Figure 3).

## Discussion

The UK broiler sector is the fourth-largest poultry meat producer in the European Union, making the United Kingdom ≈75% self-sufficient in poultry meat. The industry has benefited from the continuing consumer choice of leaner meat, reflected in the 12% rise in poultry meat sales in 2017. The widespread presence of antimicrobial resistance in *Campylobacter* spp. in retail poultry in the United Kingdom suggests horizontal transfer and mutational events within and between broiler farms and environmental conditions leading to proliferation of antibiotic-resistant lineages (*37**,**38*). This process is observed in ST5136, which was first detected in 2010 as an ST464 variant isolated from chicken meat (C0112) and clinical stool samples (ARI0509) from North East Scotland and Oxford (OXC5459). In 2012, ST5136 was reported from turkey meat (C0469) in North East Scotland, and in all cases from both years these isolates were MDR. ST5136 was never detected before 2010 in any of the 15,365 isolates submitted to the PubMLST *Campylobacter* database, but since its discovery, it has been found in >18 abattoirs across the United Kingdom. ST5136 has only ever been isolated from poultry (chicken 90%, turkey 10%, duck <1%) and never from cattle, sheep, pigs, or wild birds (*39*). Thus, human acquisition is probably exclusively from poultry meat.

The evolutionary ancestry of ST5136 shows that it is a member of CC464 and evolved from a heterogeneous complex of related strains, including ST464, all of which show high prevalence of antimicrobial resistance (Figure 3). ST5136 forms a cluster within CC464, an ancestor of CC353 with CC574 and CC354 associated more distally (Figure 2).

In the PubMLST *Campylobacter* database, the earliest report of ST354 was from a human blood culture in 1984; in 1991 it was isolated from chicken meat in the United Kingdom (*39*). ST574 was isolated in Thailand in 1999 and reported in the United Kingdom from an unspecified human sample in 2000 and chicken meat in 2001 (*39*). The closest ancestor of ST464 is ST353, which was isolated from chicken in 1982 in the United Kingdom, and ST464, reported in 2001 from chicken meat in Northern Ireland (first report of ST464 was from Germany in 2000 from human stool) and then ST5136 reported in 2010 (*39*). We compiled an illustration of the acquisition of resistance markers in ST5136 from ST464 (Figure 1). In the ancestral state, most CC464 isolates harbor *aac3* and then acquire ciprofloxacin resistance, *tet*(O/32/O) and *bla*_OXA-61_-like. Subsequently, a mutation in the *bla*_OXA-61_-like promoter leads to very high levels of ampicillin resistance. All these events have led to the stepwise selection of increasing drug resistance phenotypes in an intensely antimicrobial-competitive environment (Appendix Table 2). Ancestral to CC464 is CC353 and somewhat more distally CC574 and CC354, each of which shows progressively lower proportions of antimicrobial-resistant isolates. The commonality of the alleles for genes conferring resistance to the 4 different classes of antimicrobial drugs examined in this article indicates a gradual accumulation of these markers, culminating in ST5136. The most recently observed marker, *gyrA*_57_, is common to isolates of ST5136 and its cogenitor CC464 and leads to ciprofloxacin resistance, resulting in resistant strains that are related and are fitter than their sensitive counterparts (Figure 2; Appendix Table 2) (*34*). Because most ST5136 isolates show high levels of fluoroquinolone, β-lactam, aminoglycoside, and tetracycline resistance, it is likely that these related isolates evolved from a resistant ST464 ancestor.

The *tet*(O/32/O)_7_ and *aac3*_17_ alleles were most likely acquired previously, before the divergence of CC464 from CC353. Purifying selection of resistances could be occurring in CC464, as *tet*(O/32/O)_7_ in this group was increasingly common compared with the ancestral strains. The tetracycline resistance gene *tet*(O/32/O)_7_ is found on either plasmids or the chromosome in the ancestral isolates CC464, CC353, CC354, and CC574, but by the appearance of the CC464 ancestor of ST5136, it is found only on the chromosome, suggesting acquisition either via conjugation or by transformation into the last common ancestor. There is a constant flow of resistance genes between plasmids, phages, and bacteria in the farm environment and the chicken gut microbiome. Species such as *Streptococcus* and *Enterococcus* that form a part of the chicken gut microbiome often harbor promiscuous plasmids with resistance genes that can occur in *Campylobacter*; this exchange of genetic determinants will be a contributor to antimicrobial resistance evolution in *Campylobacter* (*40**,**41*). Similarly, OXA-193 dominated in isolates of CC464 that were closely ancestral to ST5136 and evolved to be more highly resistant to β-lactam antimicrobial drugs as a result of the –10 promoter mutation in the upstream region of *bla*_OXA-193_.

Superimposed onto this linear phylogeny of antimicrobial resistance in ST5136 are the genetic changes occurring in the other strains of the related lineages (Figure 1). In *Campylobacter* the principal sources of genetic change are intracellular mutational events and the acquisition of extracellular genes from other isolates; these events are estimated as equally common (*34**,**42*), mutations resulting in a clonal distribution of characters in related isolates and the acquisitions in a mosaic distribution of characters across the species. These sources of genetic diversity lead to the emergence of novel variants in fitter isolates selected by the environment. Thus, for *Campylobacter*, there is a complex distribution of individual genetic markers when viewed in the context of the overall phylogeny of related isolates. This finding is most clearly exemplified in the example of 1 ST5136 isolate (ARI3975), which has a basal level of resistance to ampicillin and no promotor mutation; these aspects could be the result of a spontaneous back mutation or horizontal gene transfer from a sensitive strain of *Campylobacter*.

Resistances are propagated across the clonal population of *Campylobacter*; from these survival-of-the-fittest chains of events in antimicrobial drug–rich environments, such as poultry farms, may emerge more abundant lineages due to the prophylactic or metaphylactic use of drugs (*43*). ST464 has given rise to 2 MDR strains: ST5136 (*uncA* allele 1→3) and ST5812 (*pgm* allele 10→17), which are closely associated with each other. It is unclear whether the spread of ST5136 was better facilitated than that of ST5812 or that other genetic changes in ST5136 made it more likely than ST5812 to survive and colonize in poultry or by chance.

Dissemination of highly related *Campylobacter* strains throughout the poultry industry has occurred previously. In New Zealand, *C. jejuni* ST474 (CC48) was a dominant strain found almost exclusively in isolates from 1 poultry supplier and associated with clinical cases during 2005–2008 (*44*). More recently, tetracycline- and fluoroquinolone-resistant *C. jejuni* ST6944 (CC354, one of the ancestors of CC464) has been reported in human cases and in 3 major poultry suppliers in New Zealand (*45*).

The UK poultry industry has responded well to the current BPC antibiotic stewardship program with a 71% reduction in use of antimicrobial drugs from 2012 to 2016, but it is worth noting that enrofloxacin and difloxacin are authorized fluoroquinolones currently used in the chicken industry in the United Kingdom (*46*). A 1991 study in the Netherlands reported an increase in fluoroquinolone resistance in *Campylobacter* correlated with increasing use of enrofloxacin in the poultry industry and thus the transmission of fluoroquinolone-resistant *Campylobacter* from chickens to humans (*38*). In 2012, the poultry industry phased out use of the modern cephalosporins completely, but a commitment to stop the prophylactic use of fluoroquinolones in day-old chickens was not made until 2016 (*46*). Even then, these antimicrobial drugs may be added to the drinking water of flocks of poultry when no disease is present in most of the birds in a flock (*47*). The continued occurrence of resistant strains that emerge in an antimicrobial-stressed environment but retain their resistance in environments even after the cessation of antibiotic pressure has been reported previously (*48*). Although positive antimicrobial usage may mitigate against the creation of future MDR bacteria, preexisting MDR strains, such as *C. jejuni* ST5136, may never lose their resistance characteristics.

Joshua Lederberg has said, “The future of humanity and microbes likely will unfold as episodes of Our Wits Versus Their Genes” (*49*). The evolution and rise of ST5136 indicates that bacteria can evolve by genetic adaptation to antibiotic-enriched and -deprived environments, which drives the evolution of environment-favored strains by mutation or gene transfer. It is up to us to use our wits to keep up with these changes.

Appendix Additional information about the evolution of ST5136, a multidrug-resistant strain of *Campylobacter jejuni*, Scotland.
